# The association between pregnancy-specific anxiety and fetal malformations: a cohort study

**DOI:** 10.1192/j.eurpsy.2023.1068

**Published:** 2023-07-19

**Authors:** N. Bouayed Abdelmoula

**Affiliations:** Genomics of Signalopathies at the service of Medicine, Medical University of Sfax, Sfax, Tunisia

## Abstract

**Introduction:**

Pregnancy-specific anxiety is defined as a mental state of a pregnant woman whose concerns are specific to the pregnancy itself, such as fears regarding the pregnancy, delivery, and health of the child. In the perinatal period, anxiety disorders are relatively common and are more common than depressive disorders. These psychiatric concerns can lead to long-term negative effects on pregnancy outcome. On the other hand, when the pregnancy is complicated by the diagnosis of fetal malformations, these concerns are more detrimental to maternal health.

**Objectives:**

This study aimed to determine the association between pregnancy-specific anxiety in pregnant women and the diagnosis of prenatal fetal malformations, through our database of genetic counseling and prenatal diagnoses.

**Methods:**

We conducted a retrospective study on 20 pregnant women who were referred to our genetic counselling. The consultation was piloted during a prenatal genetic exploration and counselling after the second trimester ultrasound diagnosis of syndromic and non-syndromic fetal malformations. Collection and characterization of the psychiatric concerns of the cohort were conducted using the self-reported feelings during the consultation. A conversion of these data using the Perinatal Anxiety Screening Scale was possible to assess the pregnancy-specific anxiety level of our patients.

**Results:**

Results of our study showed that pregnancy-specific anxiety is significantly associated with the discovery of fetal malformations during pregnancies. Depression, generalized anxiety, and post-traumatic stress disorder as well as a specific phobia from the malformed baby were constants as psychiatric concerns.

**Conclusions:**

Health care providers should pay special attention to pregnancy-specific anxiety. The Arabic translated version of the PASS that has been shown to have adequate validity and reliability must be used in our institutions to screen for the new concept of pregnancy-specific anxiety among all pregnant women in the perinatal phase. Women who experienced the discovery of a malformed fetus and who are facing pregnancy termination due to fetal abnormalities need to have a particular psychological support. A specific scale may be crucial in pregnant women with a malformed fetus before and after termination of the pregnancy.

**Disclosure of Interest:**

None Declared

